# Unusual severe cases of type 1 porcine reproductive and respiratory syndrome virus (PRRSV) infection in conventionally reared pigs in South Korea

**DOI:** 10.1186/s12917-015-0584-5

**Published:** 2015-10-24

**Authors:** Kwang-Soo Lyoo, Minjoo Yeom, Jong-Young Choi, Jong-Hwan Park, Sun-Woo Yoon, Daesub Song

**Affiliations:** Korea Zoonosis Research Institute, Chonbuk National University, Iksan, South Korea; Department of Pharmacy, College of Pharmacy, Korea University, Sejong, South Korea; Dodam Veterinary Clinic, Seoul, South Korea; College of Veterinary Medicine, Jeonnam National University, Gwangju, South Korea; Korea Research Institute of Bioscience and Biotechnology, Daejeon, South Korea

**Keywords:** Type 1 PRRSV, South Korea, Highly pathogenic

## Abstract

**Background:**

Porcine reproductive and respiratory syndrome virus (PRRSV) causes a loss of approximately US$ 70 million every year to the South Korean pork industry. There are two distinct genotypes: European (type 1) and North American (type 2). In South Korea, type 1 and type 2 PRRSV are widely distributed and have evolved continuously since the infection was first described. Here, we present two field cases of type 1 PRRSV infection with unusually severe pathogenicity.

**Case presentation:**

The first case farm was a two-site production system comprising farrow-to-grower and grower-to-finish units and was historically free from PRRSV infections. The PRRSV vaccine had not been used in both units. In October 2014, pigs in the grower-to-finish unit experienced severe respiratory distress with the mortality rate reaching to 22 %. Despite antibiotic treatment, clinical signs were still noticed in most pigs. The second case farm was also a two-site production system, but had two separate farrow-to-grower units (unit A and unit B). Historically, type 1 PRRSV was continuously present in unit A, but unit B was free from PRRSV. Thus, all grower pigs of unit B were vaccinated before being moved to the grower-to-finish unit. In November 2014, severe respiratory distress was seen in pigs of the grower-to-finish unit. Significant respiratory distress was observed in only the grower herd moved from unit B, and the mortality of those pigs was ~50 %. However, no disease was shown in the grower pigs from unit A.

**Conclusions:**

To our knowledge, the present study is the first observation of the cases of infection by highly pathogenic type 1 PRRSV in South Korea. The Korean type 1 PRRSV strains have undergone unique evolutionary dynamics for the last decade in this country. Although there are known to be three clusters of Korean type 1 PRRSV, their pathogenicity could not be categorized owing to their high level of genetic diversity. Therefore, further studies are needed to demonstrate the novel classification of Korean type 1 PRRSV strains according to their virulence factors.

## Background

Porcine reproductive and respiratory syndrome virus (PRRSV), a small, enveloped, and single-strand RNA virus, is a member of the family *Arteriviridae,* genus *Arterivirus* [1]. The genome of the virus is approximately 15 kb long and encodes nine overlapping open reading frames (ORFs) [1]. ORF1a and ORF 1b consist of about 75 % of the genome and encode nonstructural proteins [2]. ORF2a, ORF3, and ORF4 encode membrane-associated N-glycosylated proteins, whereas ORF2b and ORF6 encode two non-glycosylated membrane proteins [2]. ORF5 and ORF7 encode an envelope protein and a nucleocapsid protein, respectively [3]. Of these, the GP5 encoded by ORF5, which shows the highest degree of diversity within each genotype, is the major viral glycoprotein containing most of the neutralizing epitopes and is involved in many important disease processes [3–5].

The disease caused by PRRSV is characterized by respiratory distress in nursery and growing pigs as well as reproductive failure in breeding-age pigs, and causes approximately US$ 70 million in losses every year to the South Korean pork industry estimated by the assessment of the economic impact on the US pork industry [6]. Genetically, PRRSV has been revealed to be of two distinct genotypes: European (type 1) and North American (type 2), both of which have significant differences in terms of clinical and antigenic aspect [7]. In South Korea, type 2 PRRSV has evolved continuously and has been subsequently characterized into at least four lineages, ever since the infection by this PRRSV genotype was first described in 1993 [8]. Recently, it was revealed that type 1 PRRSV to be not only widely distributed in South Korea but also divided into three genetic clusters based on phylogenetic analysis [9].

Although both genotypes of PRRSV can raise similar clinical signs in pigs, type 2 PRRSV has been demonstrated to have significantly greater virulence to induce respiratory disorder than type 1 PRRSV [10, 11]. Martínez-Lobo et al. [12] demonstrated that pigs infected with the type 2 PRRSV strain showed more severe respiratory clinical signs and macro- and microscopic lung lesions than pigs inoculated with the type 1 PRRSV strain. van der Linden et al. [13] confirmed the difference in kinetics of the immune responses between type 1 and type 2 PRRSVs and more severe clinical signs in pigs infected with a type 2 strain than with type 1 strains. In contrast, the East European subtype 3 of PRRSV strain “Lena”, which has antigenic heterogeneity with other type 1 or type 2 strains, showed pathogenic severity in conventional pigs [14]; however the highly pathogenic type 1 strain has not been reported in South Korea.

In this study, we present two field cases of type 1 PRRSV infections at swine farms with unusually disease severity compared with what has been previously reported for type 1 PRRSV-infected pigs in South Korea. The monitoring for both farms was performed by the attending veterinarian who consistently recorded the farm’s history and biopsied the samples during the outbreak.

## Case presentation

### Case 1

The case farm was a two-site production system (i.e., a farrow-to-grower unit with 400 sows and a grower-to-finish unit) in central South Korea. The grower-to-finish unit was located 10 km east of the sow farm. The sow farm was historically free from infections with PRRSV, *Mycoplasma hyopneumoniae*, and porcine circovirus type 2 (PCV-2), and PRRSV vaccine had not been used in both units. In October 2014, the grower-to-finish unit experienced an outbreak of acute respiratory disease in the approximately 8-weeks-old grower pigs. The onset of the disease was observed at about 3 weeks after the arrival of the pigs, and whereupon they exhibited severe respiratory signs including high fever (41–42 °C), dyspnea, coughing, and emaciation. When the mortality rate reached 22 %, the herd veterinarian prescribed Florfenicol administration in water and Marbofloxacin by intramuscular injection into the pigs. Despite the antibiotics treatment and a decrease in mortality, clinical signs were still noticed in most pigs. For the laboratory diagnosis, lung samples collected from autopsied pigs were sent to the Virology Laboratory, College of Pharmacy, Korea University, Sejong, South Korea.

### Case 2

The second case farm was also a two-site production system (viz., farrow-to-grower units and a grower-to-finish unit) in northwestern South Korea. The farm had two separated farrow-to-grower units (unit A with 400 sows and unit B with 200 sows) with a distance of 25 km between them. The individual grower-to-finish unit was located 20 km south of the farrow-to-grower units. Historically, PRRSV (type 1) was continuously present in unit A, and a high level of antibody titer to PRRSV was maintained within those pigs. In contrast, unit B was free from PRRSV infection. Thus, all pigs (5-week-old) of unit B were vaccinated with a live attenuated type 1 PRRSV vaccine (Porcilis® PRRS, MSD) before being moved to the grower-to-finish unit. On the other hand, no vaccination for PRRSV was conducted on the grower pigs of unit A. In November 2014, the same acute and severe respiratory disease as seen in the Case 1 farm was observed in pigs (11-weeks-old) of the grower-to-finish unit upon 3 weeks of their arrival. Interestingly, the significant respiratory distress was observed in only the grower herd moved from unit B, and the mortality of those pigs was ~50 %. However, no disease was evident in the grower pigs from unit A, even though the grower pigs with (from unit B) or without (from unit A) respiratory symptoms were housed in adjacent pens in the same barn. The herd veterinarian suspected a PRRSV infection and collected lung tissues from autopsied symptomatic pigs for laboratory diagnosis.

## Laboratory examinations

### Pathologic findings

**Fig. 1:**
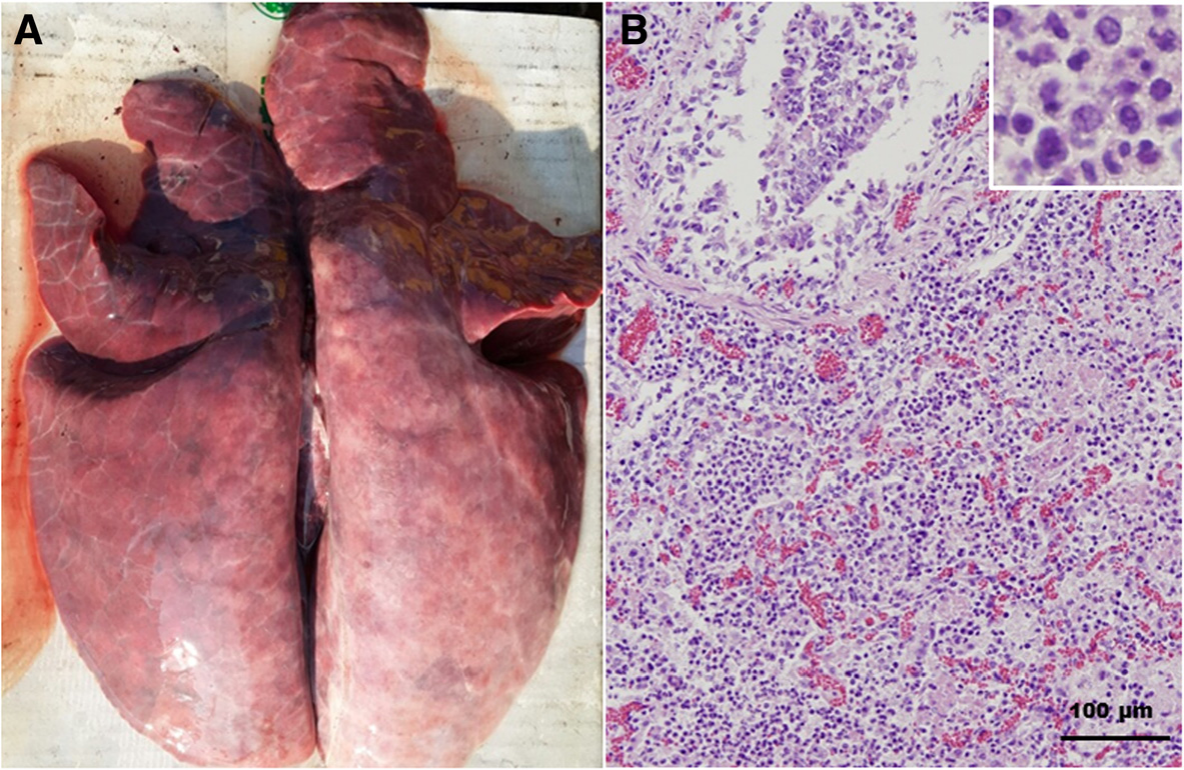
Gross lesion and histopathologic lesion of a pig from the first case farm affected infected by type 1 PRRSV infection. Gross lung lesion was characterized by diffusely mottled, red to purple, and rubbery areas of pneumonia (**a**). Histologically, purulent bronchopneumonia by infiltration of monocytes, lymphocytes, and neutrophils and sever congestion was seen in the lung (**b**)

At necropsy, the lungs of affected pigs from the two case farms failed to collapse and were diffusely mottled, red to purple, and rubbery (Fig. 1a). Tissue samples from each lung lobe were fixed in 10 % neutral buffered formalin overnight at 4 °C, dehydrated in a graded ethanol series, embedded in paraffin wax, sectioned at 3 μm thickness and stained with hematoxylin and eosin reagent. Histopathologically, purulent bronchopneumonia that is characterized by infiltration of monocytes, lymphocytes and a few number of neutrophils and sever congestion was seen in the lungs. The inflammatory cells were infiltrated in alveolar spaces and walls and sometimes in the lumen of bronchioles, which resulted in destruction of the lung parenchyma (Fig. 1b). Written informed consent was obtained from the farm owners for the publication of the evaluation data and any accompanying images.

### Molecular findings

Lung tissue samples were collected from the necropsied pigs, and RNAs were extracted from the lung tissue suspension (20 % in PBS) using QIAamp viral RNA mini kit (Qiagen) according to the manufacturer’s manual. The cDNA was synthesized using random primer and a commercial M-MLV reverse transcriptase kit (Invitrogen) according to the manufacturer’s manual. To sequence the ORF5 gene for type 1 and type 2 PRRSVs, nested PCR was performed as previously described [8]. The amplified ORF5 gene fragments of type 1 PRRSV were purified using the QIAquick Gel Extraction kit (Qiagen); however, the gene for type 2 PRRSV was not detected in any of the samples. Conventional PCR and RT-PCR were performed to rule out other suspected pathogens, including PCV-2, classical swine fever virus, swine influenza virus, *Actinobacillus pleuropneumoniae*, *Pasteurella multocida* in the lung samples according to the methods described previously [15–18], and the test results were negative for the pathogens in all samples.

Analysis of the nucleotide sequences of the ORF5 gene of the type 1 PRRSV isolates was performed using BLAST/Align (bl2seq) on the NCBI website and the MEGA (ver.4.1) program. Nucleotide sequences of the ORF5 genes of the isolates were aligned using the ClustalX (ver. 1.81) software, and a phylogenetic tree was constructed by the neighbor-joining method with ORF5 genes of other PRRSV strains from GenBank. The phylogenetic distances were determined by bootstrapping with 1000 repeats and molecular evolutionary analysis was performed using the MEGA (ver.5.0) program [19].

**Fig. 2:**
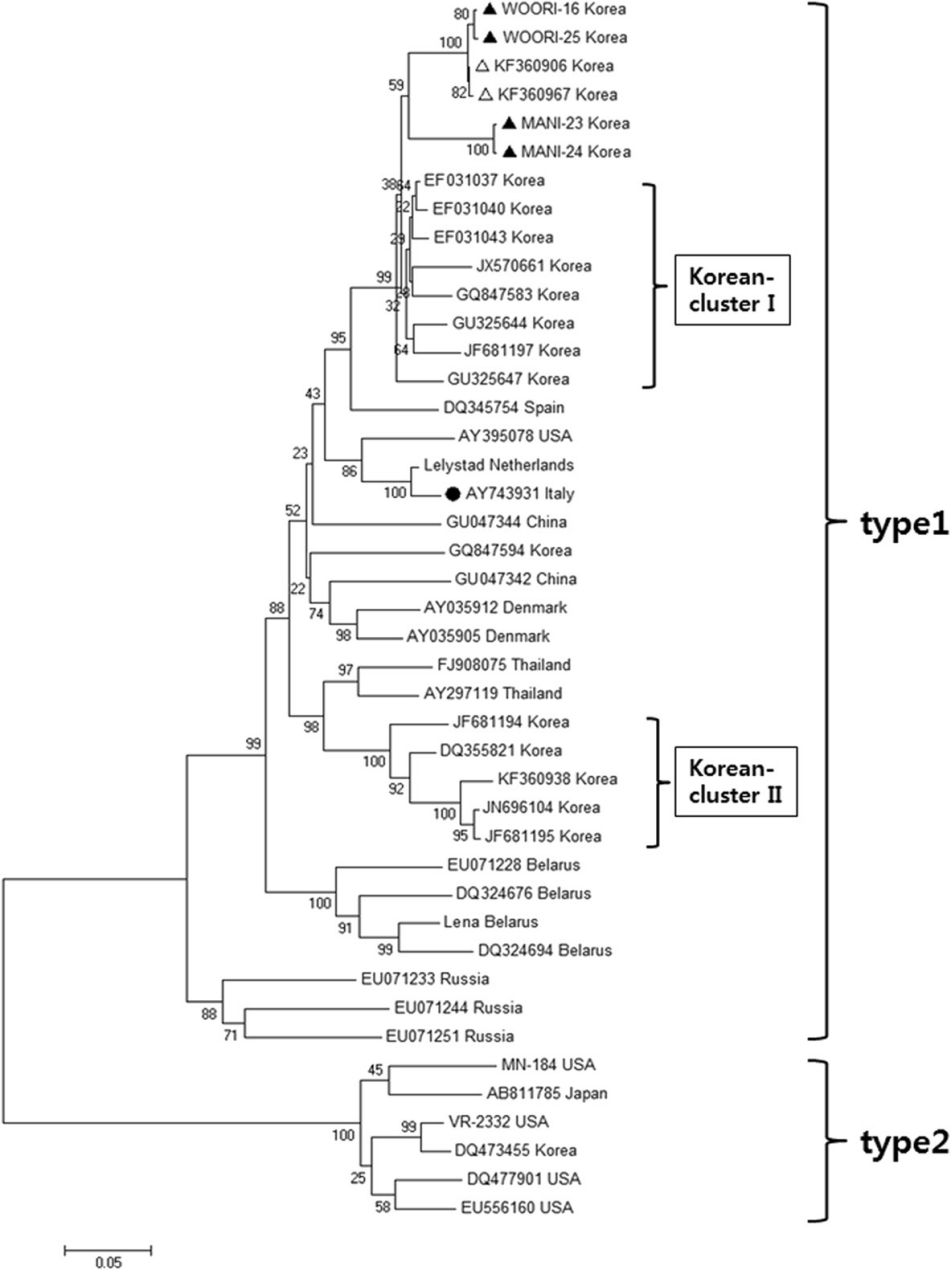
Phylogenetic analysis of type1 and type 2 PRRSV strains including Korean isolates based on the nucleotide sequences of the complete ORF5 genes. The branches indicate bootstrap values calculated from 1,000 bootstrapping replicates. ▲: PRRSV isolates identified in this study. △: the most similar strains with the isolates in this study. ●: the vaccine strain used in case 2

In comparison among nucleotide sequences of ORF5, type 1 PRRSV isolates WR-16 and WR-25 (Accession No.: KT257724 and KT257725) from the first case farm and isolates MANI-23 and MANI-24 (Accession No.: KT257726 and KT257727) from the second case farm exhibited 86.3–86.6 % homology with European prototype PRRSV strain “Lelystad (LV)”, whereas cluster I strains of Korean type 1 PRRSV showed higher homology (88.4–89.9 %) in with strain LV . In the phylogenetic analysis based on the ORF5 gene, the isolates from both case farms were closely related and belonged to cluster I of Korean type 1 PRRSV, although it seems that the isolates identified in the current study generated a sub-cluster within cluster I with two other Korean type 1 strains (Accession No.: KF360906 and KF360967) (Fig. 2).

## Discussion

Ever since type 1 PRRSV was first observed in South Korea in 2005, the virus has distributed nationwide and evolved unique genetic properties with a high degree of variation [9]. In experimental pig studies, the pathogenicity of Korean type 1 and type 2 PRRSV strains was compared on the parameters of viral loads in blood, pathologic scores of lungs and lymph nodes, and viral shedding level in pigs, where the type 2 showed greater virulence than type 1 [20]. On the contrary, another Korean type1 PRRSV strain induced more severe gross lesions in lungs than a type 2 PRRSV strain (VR-2332-like) [21], whereas similar pathogenicity between both genotypes of Korean PRRSV strains was shown in experimentally infected boars [22]. Thus, it could be presumed that Korean type 1 PRRSVs have a broad range of virulence in pigs, although comprehensive data for the comparative pathogenicity between type 1 and type 2 isolates are still lacking. Nevertheless, field observation of highly pathogenic type 1 PRRSVs has not been reported to our knowledge.

Recently, Nguyen et al. [23] performed a broad phylogenetic investigation with the ORF5 sequences of about 1100 isolates worldwide reported in GenBank. They speculated that the evolutionary dynamics of Korean type 1 PRRSVs is unique in terms of genetic epidemiology on the ORF5 gene, and suggested that there are two major clusters (cluster I and cluster II) of type 1 PRRSV currently circulating in South Korea [23]. In the present study, the isolates from the pig farms with severe respiratory problems were regarded as Korean cluster I strains through phylogenetic analysis. Because there is a wide range (88.9–100 %) of nucleotide sequence diversity for the ORF5 gene within Korean cluster I [23], it seems that the pathogenicity associated with the diversity would be various in pigs. Therefore, it is hard to conclude which genetic category can represent the pathogenicity of each type 1 strain. Notably, the genetic diversity between strain LV and the isolates considered as highly-pathogenic type 1 PRRSVs was higher than that between strain LV and the Korean type 1 PRRSV cluster I strains. Because there were limited data to phylogenetically classify another cluster or sub-cluster for the isolates of the current study, more investigation comprising farm histories and disease pathogenicity needs to be carried out for establishing the epidemiologic dynamics of Korean PRRSV strains.

Isolates WR-16 and WR −26 showed over 99 % identity with two isolates reported by other Korean researchers. Unfortunately, although we tried through personal communications to acquire the farm histories of these other two strains, it could not be confirmed whether they also caused severe respiratory disease in the farms. In fact, it is speculated that some strains of Korean type 1 PRRSV have probably caused severe respiratory diseases in pig farms in South Korea since the genotype was first transmitted into this country. However, a differential diagnosis for type 1 PRRSV outbreaks without type 2 PRRSV infection in conventionally reared pigs may be difficult for severe respiratory failure, as type 2 PRRSVs are epidemic nationwide.

Interestingly, in the second case farm, growing pigs vaccinated with a commercial modified attenuated live type 1 PRRSV vaccine before transfer were infected by type 1 field PRRSV and showed significant respiratory symptoms and high mortality, whereas other growing pigs from the farrow-to-grower herd that was endemically infected with type 1 PRRSV were healthy and without any clinical signs. This disease occurrence was completely unexpected by the farm owner and the veterinarian, because it has been demonstrated that the commercial vaccine (Porcilis® PRRS, MSD) based with type 1 PRRSV is effective against PRRSV infection by not only pan-European subtype 1, but also East European subtype 3 [24, 25]. In an experimental challenge study by a Korean research group, growing pigs vaccinated with the vaccine were protected against infection by a Korean type 1 PRRSV strain (pan-European subtype 1-like); however the lack of cross-protection against type 2 PRRSV challenge was observed [26]. Nonetheless, it is assumed that the commercial vaccine would not provide protective efficacy against some strains of type 1 PRRSVs that are currently circulating in South Korea.

## Conclusions

To our knowledge, the present findings are the first observation of cases of infection by a highly pathogenic type 1 PRRSV in conventionally reared pigs in South Korea. Although Korean type 1 PRRSV strains have undergone unique evolutionary dynamics for the last decade in this country, the observation of severe respiratory disease by a single infection with type 1 PRRSV has not been verified because the disease problems from type 2 strains are more common in South Korea. Moreover, it was also essential that the case farms had to have been monitored by a veterinarian for a long period for those cases to be recognized. Although two clusters based on the ORF5 gene of Korean type 1 PRRSVs have been previously demonstrated, the pathogenicity of the Korean isolates could not be categorized due to their high level of genetic diversity. Therefore, further studies are needed to demonstrate the novel classification of Korean type 1 PRRSV strains according to their virulence factors of the virus.

## Abbreviations

PRRSV: Porcine reproductive and respiratory syndrome virus
ORF: Open reading frame
PBS: Phosphate buffered saline
PCV-2: Porcine circovirus type 2
RT-PCR: Reverse transcription polymerase chain reaction
